# Extrinsic role of gibberellin mitigating salinity effect in different rice genotypes

**DOI:** 10.3389/fpls.2022.1041181

**Published:** 2022-10-25

**Authors:** Muhammad Farooq, Muhammad Aaqil Khan, Dan-Dan Zhao, Saleem Asif, Eun-Gyeong Kim, Yoon-Hee Jang, Jae-Ryoung Park, In-Jung Lee, Kyung-Min Kim

**Affiliations:** ^1^ Department of Applied Biosciences, Graduate School, Kyungpook National University, Deagu, South Korea; ^2^ Crop Foundation Research Division, National Institute of Crop Science, Rural Development Administration, Wanju, South Korea

**Keywords:** Cheongcheong, Gibberellin, IR28, OsAmy1A, OsAmy1C, OsAmy3C

## Abstract

The overall effects of gibberellic acid (GA3) with NaCl on different rice genotypes are inadequately understood. The present study determines the effect of different GA3 (50 and 100 µM) concentrations on the morphophysiological, molecular and biochemical effects of 120 mM NaCl salt stress in rice seedlings. Salt stress reduced germination percentages and seedling growth and decreased bioactive GA content. It also downregulated the relative expression of α-amylase-related genes – *OsAmy1A*, *OsAmy1C*, and *OsAmy3C* in the salt-sensitive IR28 cultivar. Salt stress differentially regulated the expression of GA biosynthetic genes. Salt stress increased antioxidant activity in all rice genotypes tested, except in IR28. GA3 mitigates the effect of salt stress, rescuing seed germination and growth attributes. GA3 significantly increased bioactive GA content in Nagdong and pokkali (50 µM) and Cheongcheong and IR28 (100 µM) cultivars. The α-amylase genes were also significantly upregulated by GA3. Similarly, GA3 upregulated *OsGA2ox1* and *OsGA2ox9* expression in the Cheongcheong and salt-sensitive IR28 cultivars. The present study demonstrated that salt stress inactivates bioactive GA – inhibiting germination and seedlings growth – and decreases bioactive GA content and GSH activity in IR28 and Pokkali cultivars. Further, GA3 significantly reversed the effects of 120 mM NaCl salt stress in different rice genotypes. The current study suggested that the known coastal area salinity concentration can be significantly recovered with the application of exogenous GA3. Thus, it could be possible to grow eco-friendly rice close to the coastal zone in order to reduce the damage caused by salinity.

## Introduction

Abiotic stress salinity significantly affects the agricultural production of rice (*Oryza sativa L.*) worldwide. One estimate is that relatively 6 and 20% of barren and irrigated land, are usually affected by salinity (Munns and Tester, 2008). The range of saline agricultural land is increasing more and more, particularly because of high-salt irrigation water and unsuitable irrigation management techniques (Plaut et al., 2013). Salt stress prevents various physiological processes, such as seed germination, and growth attributes decrease the rate of photosynthesis, accelerate senescence, and reduce rice production (Tuteja et al., 2013).

**Figure 1:**
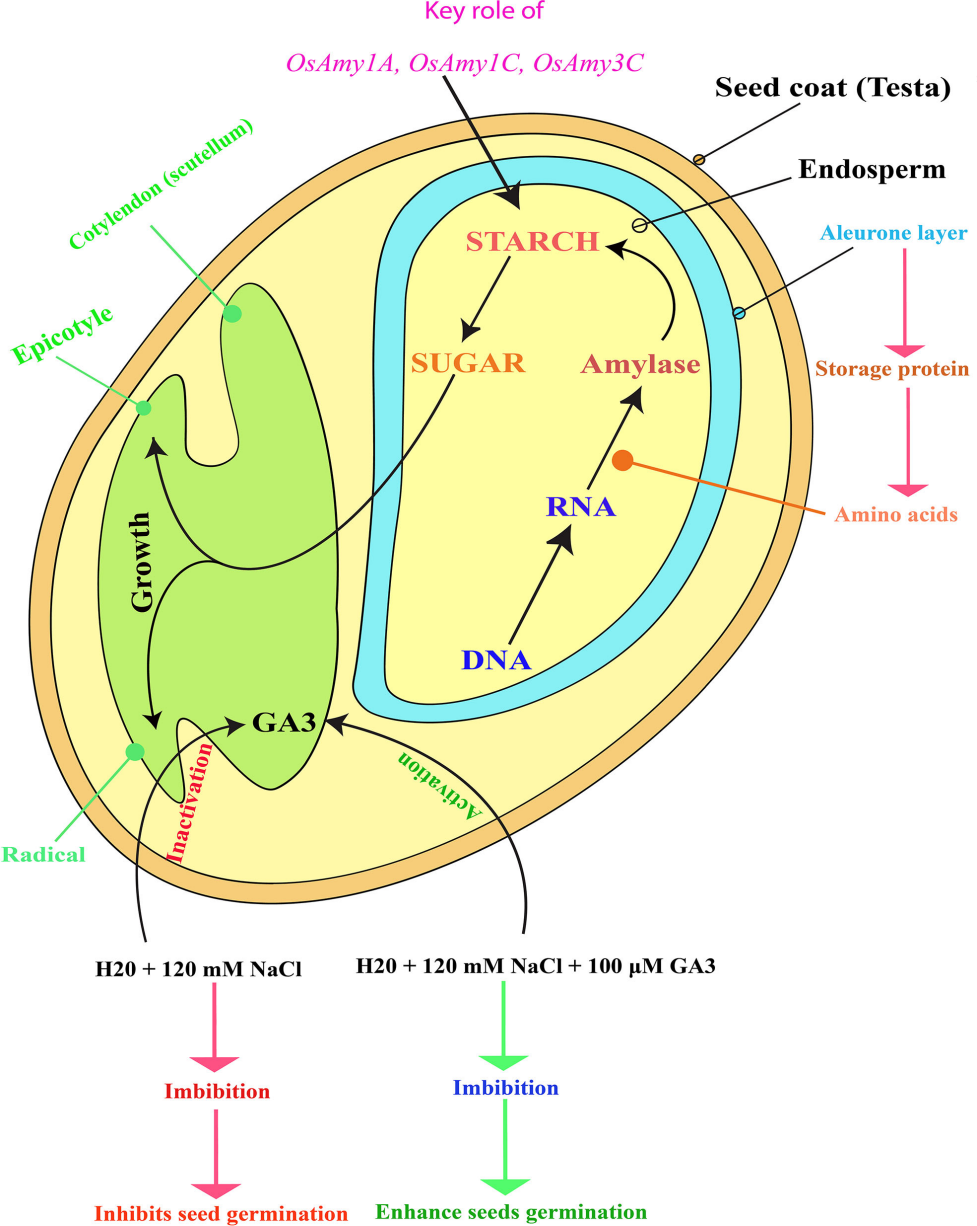
Schematic representation of rice (*Oryza sativa* L.) seed structure, depicting how salinity stress inactivates endogenous GA3, leading to the inhibition of both rice seed germination and seedling growth. Figure adapted from (Koning, 1994; Ma et al., 2017). Exogenous GA3 application reactivates endogenous GA3, enhancing rice seed germination and seedling growth. GA3 activation also upregulates the α-amylase genes (*OsAmy1A*, *OsAmy1C*, and *OsAmy3C*) that may help to degrade starch contents and convert them into sugar. Later, this sugar serves as food for young plants (embryos) in which seed germination and seedling growth is restored. In the diagram, the red color letter indicates the suppression of seed germination and inactivation of GA3, whereas the green color represents the stimulation of seed germination and activation of GA3. The pink color indicates the activation of the α-amylase genes, which prevented seed germination due to the saline effect.

Germination is the process that begins when dormant dry seeds gain water and ends with the termination of innate axis elongation (Bewley and Black, 2013). Under salinity stress, exogenous brassinosteroids show an important role in recovering the inhibitory effects of this stress on seed germination and seedling growth (Anuradha and Rao, 2001). Salinity stress of 12 dS m^-1^ (decisiemens per meter) decreases various physiological processes in maize such as reducing the length of root and shoot, fresh and dry weights, chlorophyll and carotenoid contents, K^+^ ion accumulation, and affects the activities of antioxidant enzymes while enhancing oxidative damage and high Na^+^ accumulation, however, the exogenously applied GA3 improved their growth attributes, decrease oxidative stress, increased K^+^ ion content, induces antioxidant genes expression and activities (Shahzad et al., 2021). The application of GA3 during seed priming increases water absorption, metabolic activity, and emergence time of seeds (Raj and Raj, 2019). A low concentration, of GA3, breaks seed dormancy and increases plant growth and overall productivity (Pawar and Laware, 2018). There may be inter-linked activities between ABA and GA as, in both the *aba* and *rod2* mutants of *Arabidopsis*, decrease dormancy occurs concurrently with a dropped GA threshold required to achieve germination (Léon-Kloosterziel et al., 1996). GA and ABA are two major hormones with antagonistic effects on germination (Llanes et al., 2016; Shu et al., 2016). A previous study testified that salinity inhibits germination in soybean seed by decreasing the GA/ABA ratio *via* decreased bioactive GA and increased ABA content (Shu et al., 2017). Germination process is regulated by several internal and external factors such as phytohormones, temperature, and light (Qu et al., 2008; Weitbrecht et al., 2011). Salt stress generally inhibits seed germination, whereas GA can be promotes it (Gill et al., 2002; Chang et al., 2010). In rice, seed germination is significantly inhibited by salt stress and this inhibition can be alleviated by GA (Liu et al., 2018) (**Figure 1**).

Plant hormones including GA, ABA, IAA, ethylene, cytokinin, and brassinosteroids, are biochemical substances that regulate various physiological and biochemical processes in plants. These fascinating products are synthesized by plants and soil microbes (Finkelstein, 2004; Jiménez, 2005; Santner et al., 2009). The phytohormones gibberellin is a group of tetracyclic diterpenoids that regulates many developmental processes, together with stem elongation, seed germination, dormancy, flower development, flowering, and leaf and fruit senescence (Hedden and Sponsel, 2015). GA regulation is mainly affected by GA metabolism, including its biosynthesis and inactivation (Hedden and Phillips, 2000). Currently, there are 126 known pure GA compounds that have been derived and characterized from natural sources. Among these, only GA1, GA3, GA4, and GA7 are considered the bioactive forms of GA in higher plants. Various enzymes and their respective genes catalyse GA biosynthesis, including *ent-copalyl* diphosphate synthase (*CPS*) ent-kaurene synthase (*KS*), ent-kuarene 19-oxidase (*EKO*), GA_12_-aldehyde 7-oxidase (*GA7ox*), GA20-oxidase (*GA20ox*), GA3ox-hydroxylase (*GA3ox*), and GA 2-oxidase (*GA2ox*) (Hedden and Phillips, 2000).

There are some enzymatic and non-enzymatic antioxidants that play a vital role in minimising or alleviating high ROS accumulation in a well-organized plant system (Apel and Hirt, 2004; Karuppanapandian et al., 2011). Enzymatic antioxidants comprise catalase (CAT), peroxidase (POD), superoxide dismutase (SOD), along with the enzymes of the ascorbate (ASC)- glutathione (GSH) cycle that reclaim ROS: GSH reductase; ASC peroxidase, monodehydroascorbate dehydrogenase; and dehydroascorbate reductase (Foyer and Noctor, 2011; Chawla et al., 2013). Non-enzymatic antioxidants include ASC, flavonoids, GSH, phenolics, and tocopherols (Munné-Bosch, 2005; Gupta and Huang, 2014; Rakhmankulova et al., 2015; Talbi et al., 2015). Many reported studies have suggested that plants are well equipped with a diverse array of antioxidant enzymes to protect against oxidative damage by ROS, such as CAT, SOD, POD, and polyphenol oxidase (Dat et al., 2000; Vaidyanathan et al., 2003; Agarwal and Pandey, 2004).

Rice is one of the most important staple foods for over half of the world’s population (IRR) and has an impact on the livelihoods and economies of several billion people. In recent years, salinity stress has become a major driver of decreasing rice growth and yield worldwide, particularly in South Korea. Therefore, in the current study, we used two famous rice cultivars, Cheongcheong and Nagdong, from a plant molecular breeding lab (South Korea), and two additional cultivars, the salt-sensitive IR28, and salt-tolerant Pokkali. This study determined the effect of salt stress on these rice genotypes, with or without exogenous GA3 application. Furthermore, the GA metabolism, effects of salt on GA metabolism, expression of α-amylase and GA biosynthesis-related genes, and antioxidant activity were investigated in the different rice cultivars in this study.

## Materials and methods

### Plant materials and growing conditions

Seeds from four different rice (*Oryza sativa L.*) cultivars, Cheongcheong, Nagdong, IR28, and Pokkali (Gyehwa-20) were used in this study. Cheongcheong and Nagdong are two well-known rice cultivars generally used in South Korea. Pokkali is a one of unique salt-resistant cultivar that is cultivated worldwide in waterlogged and seaside areas, and IR28 is a salt-sensitive cultivar. The rice genotypes Cheongcheong and Nagdong were used for evaluation in this study. Seeds of all rice genotypes surface sterilised with 70% ethanol, 5% sodium hypochlorite, for 1 and 20 min. Sterilised clean seeds were germinated in Petri dishes (9 cm in diameter) according to the method described by (Liu et al., 2018) with slight modifications, treated with 30 ml distilled water (control), 120 mM NaCl, 120 mM NaCl + 50 µM GA3 or 120 mM NaCl + 100 µM GA3. All seeds were germinated in a growth at 28°C for 2 weeks.

### Seed germination and growth analysis

Germination rates were calculated after 7 and 14 days. Each experimental condition had four replicates and each replicate included 45 seeds. Experiments were repeated three times for each treatment. Seed germination was considered to occur when the radicle protruded from its primary root. To determine rice seedling growth, 20 seedlings of each cultivar were randomly selected from four replicates, and seedling length measured in cm.

### Quantitative real-time PCR analysis

Seeds of each rice cultivar were grown in double-distilled water in Petri-dishes containing an autoclaved filter paper. After 14 days, seedlings were treated with 15 ml of distilled water (control group), 120 mM NaCl, 120 mM NaCl + 50 µM GA3, or 120 mM NaCl + 100 µM GA3; three replicates were performed for each cultivar per condition. After 6 h, the roots and shoots samples were collected and RNA extracted from the root and shoot regions using RNeasy Plant Mini Kit (Qiagen, Germany) the manufacturer’s guidelines. A qPCRBIO cDNA Synthesis Kit was used for first-stand cDNA synthesis, using 500 ng total RNA. Quantitative real-time PCR (qRT-PCR) was performed using the Steponeplus Real-time PCR System (Life Technologies Holdings Pte. Ltd., Singapore), Biofact 2x Real-Time PCR Master Mix (including SYBR Green I) (Biofact, South Korea), and primers specific to the selected genes (**Table 1**). *OsActin1* from our previous work with (accession no. AB047313) was used as the internal control gene for normalization.

**Table 1:** Primer list for quantitative real-time PCR analysis.

Accession No.	Gene	Forward primer (5′-3′)	Reverse primer (5′-3′)
LOC_Os02g52710	*OsAmy1A*	ACAAGGTCATGCAGGGCTAC	AGATCGCTGTCAGCTTCCAT
LOC_Os04g5270	*OsAmy1C*	ACGACCATTTCTTCGACTGG	CGACCTTCGTGATGACCTTT
LOC_Os08g36900	*OsAmy3C*	CACGACAAGAACGTCGAGTG	TTGGAGTACTGCGTGTCGTC
LOC_Os02g17780	*OsCPSI*	GCAATGTTTTGGGGCTAGAA	TCATCCATTCAATCCAAGCA
LOC_Os04g52230	*OsKSI*	ACCTGTTGGAGTGGGATGAG	ATCCACCCATGAAAGCTGAC
LOC_Os06g02019	*OsKAO*	CTGTGCCCTGGAAATGATCT	ATCGGAAACTTTGGTGATCG
LOC_Os05g06670	*OsGA2ox1*	ACCACTACCCTCCATCATGC	CACCTGTAGCCCTTCCACAT
LOC_Os01g55240	*OsGA2ox3*	GCGACTCCTTCTTCGTCAAC	TGGTGCAATCCTCTGTGCTA
LOC_Os02g41954	*OsGA2ox9*	GTACAAGAGCGTGGAGCACA	CCGTCTTCTTGACGTCTTCC

### Measurement of bioactive GA content in rice seedlings

Seeds from the four different rice genotypes were grown for 14 days in a growth room, in either distilled water (control group), 120 mM NaCl, and treated with 50 or 100 µM GA3. Subsequently, rice seedlings were collected and immediately ground in liquid nitrogen using a pestle and mortar, Endogenous GAs were quantified described by (Lubna et al., 2022).

### Measurement of GSH and POD activity

Rice cultivars were grown for two weeks at 28°C in Petri dishes containing distilled water (control), 120 mM NaCl, and with 50 or 100 µM GA3 treatment. Seedlings samples were collected after 14 days, and 200 mg (frozen weight; FrW) frozen tissue samples were ground immediately in a liquid nitrogen; samples were then homogenised in 2 ml 10% trichloroacetic acid. After centrifugation at 10,000 × g for 15 min, 0.1 ml supernatant was moved into 3 ml 150 mM monosodium phosphate buffer and 0.5 ml Ellman’s reagent and incubated at 30°C for 5 min. Relative absorbance was then measured spectrophotometrically at 420 nm. For POD activity briefly, 500 mg seedlings samples were ground in liquid nitrogen. Following this, 0.1 M potassium phosphate buffer (pH 6.8) was added to the samples, which were then centrifuged at 4°C for 15 min, at 5000 rpm. A reaction mixture containing 0.1 M potassium phosphate buffer (pH 6.8), 50 µl pyrogallol (50 µM), and 50 µl H_2_O_2_ (50 µM) was mixed with 100 µl sample crude extract. After incubating this reaction mixture at 25°C for 5 min, 5% H_2_SO_4_ (v/v) was added to stop the enzymatic reaction. The resulting absorbance was measured spectrophotometrically at 420 nm.

### Statistical analysis

Group differences were analysed using an independent sample one-way or two-way ANOVA, followed by Turkey’s multiple comparisons test, with at least three replicates. p values < 0.05 were considered statistically significant. All data are expressed as the mean ± SE.

## Results

### Exogenous GA3 enhanced seed germination and growth in rice under salt stress

**Figure 2:**
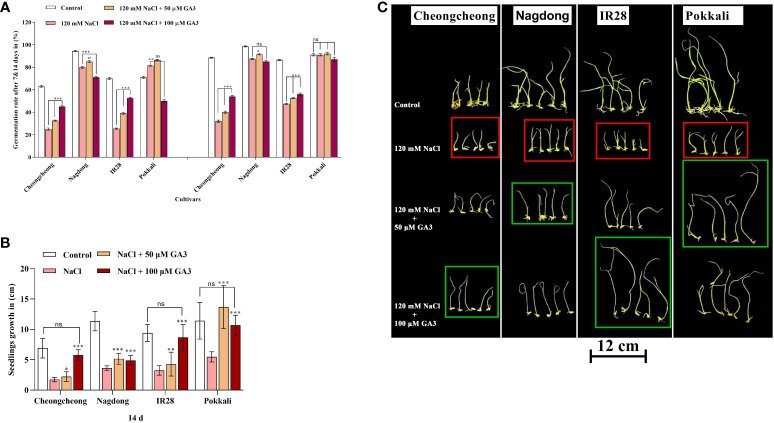
Effect of salt stress and exogenous GA3 application on seed germination in different rice (*Oryza sativa* L.) cultivars are indicated in red, and green boxes. The effects of different concentrations of exogenous GA3 on seed germination **(A)**, seedling growth **(B)**, and seedling phenotypes **(C)** were evaluated in rice cultivars under salt stress. Group differences were analysed by two-way ANOVA. Asterisks indicate a significant difference compared to the control group, as analysed by Duncan’s multiple comparison test. *P < 0.05, **P < 0.01, ***P < 0.00; ns, not significant. GA3, gibberellic acid.

Seed germination in rice genotypes under salt stress and treated with exogenous GA3 was analysed at 7 and 14 d. Salt stress drastically reduced the seed germination percentage in both Cheongcheong and IR28 cultivars compared to the distilled water controls, whereas Nagdong and Pokkali cultivars responded well to salinity (**Figure 2A**). Exogenous GA3 rescued seed germination by 15–20% in both the Cheongcheong and salt-sensitive IR28 cultivars (**Figure 2A**); notably 100 µM GA3 significantly increased the germination percentage in both cultivars compared to the salt stress-only condition. Although the Nagdong and Pokkali cultivars were more salt-tolerant under 120 mM NaCl salt stress, 50 µM exogenous GA3 slightly enhanced seed germination in these cultivars, an effect not seen with 100 µM GA3. Seedling growth in all rice genotypes significantly decreased under 120 mM NaCl stress compared to that of distilled water controls (**Figure 2B**); both concentrations of GA3 tested were able to enhance seedling growth under salt stress. Seedlings from the Nagdong and salt-tolerant Pokkali cultivars responded well to 50 µM GA3 under 120 mM NaCl stress, an effect slightly diminished at 100 µM GA3 (**Figures 2B, C** **)**. In contrast, seedling growth in the salt-stressed Cheongcheong and salt-sensitive IR28 cultivars significantly increased with both 50 and 100 µM GA3 treatment. However, 100 µM GA3 more effectively enhanced seedling growth in the Cheongcheong and salt-sensitive IR28 cultivars (**Figures 2B, C** **)**.

### Exogenous GA3 rescued the salt stress-induced bioactive GA deficiency in rice seedlings

**Figure 3:**
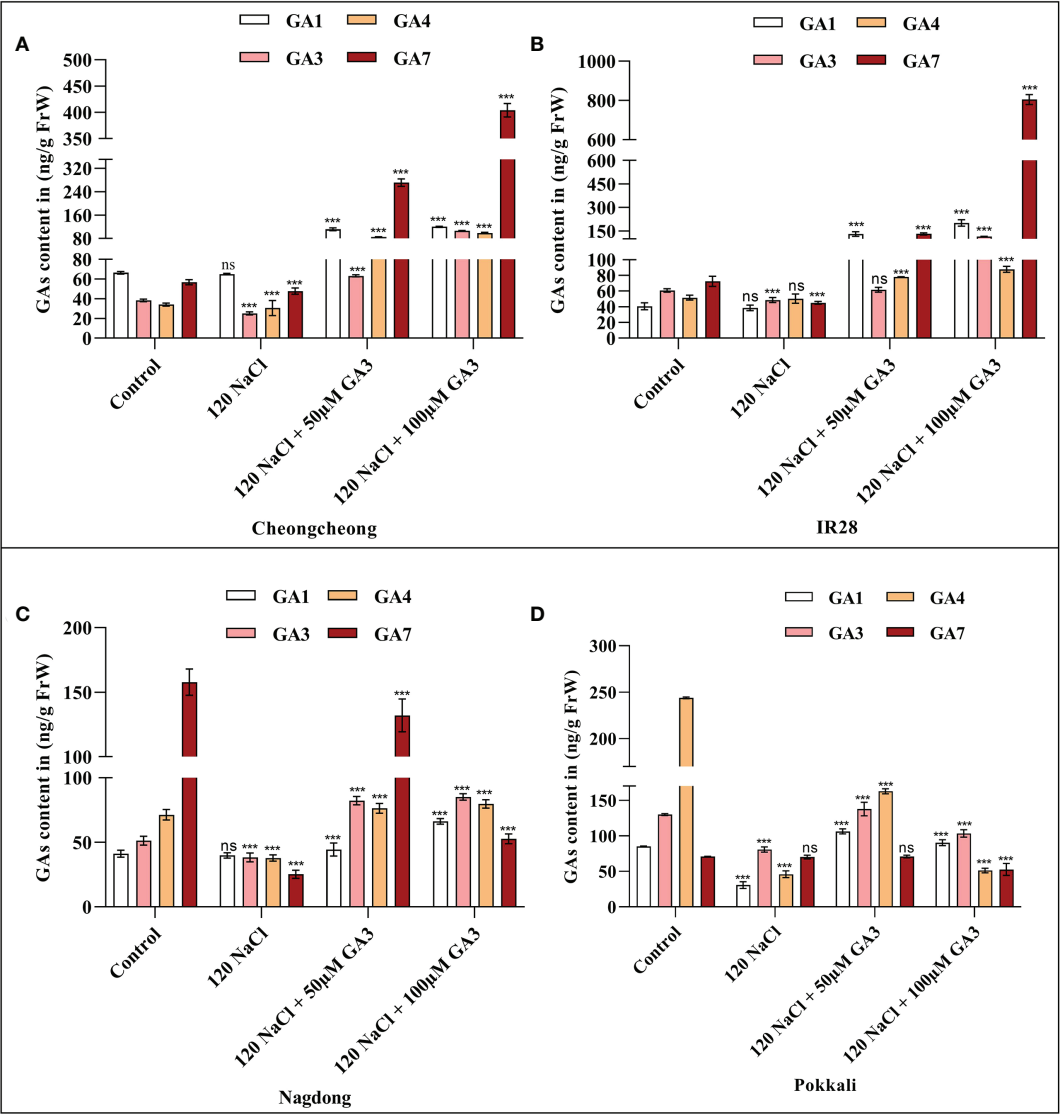
GA quantification in 14-day old rice (*Oryza sativa* L.) seedlings, treated with distilled water (control), 120 mM NaCl, 120 mM NaCl + 50 µM GA3, or 120 mM NaCl + 100 µM GA3 **(A–D)**. Group differences were analysed by two-way ANOVA. Asterisks indicate a significant difference compared to the control group, as analysed by Tukey’s multiple comparisons test. ***P < 0.00; ns, not significant. GA, gibberellin; GA1, gibberellin A1; GA3, gibberellic acid; GA4, gibberellin A4; GA7, gibberellin A7.

GA1 and GA4 are considered the essential bioactive forms of GA in rice. Generally, salinity stress decreases the bioactive GA content in germinating seeds. As we observed an effect of both 120 mM NaCl, and 50 or 100 µM exogenous GA3 on different rice seeds, we measured the levels of bioactive GAs, namely GA1, GA3, GA4, and GA7, under these conditions. To first examine whether the bioactive GA content was decreased by salinity, the levels of GA1, GA3, GA4, and GA7 in rice seedlings were determined after 14 days. NaCl treatment decreased the level of bioactive GAs in different rice seedlings compared to those in the distilled water controls (**Figures 3A** **–D**). We observed that 120 mM NaCl stress significantly decreased t the bioactive GA content in seeds from all rice genotypes. These results suggest that 120 mM NaCl salinity stress causes a deficiency in bioactive GAs in these rice genotypes that may inhibit seed germination and seedling growth. We observed that GA3 improved the effects of salt stress, with 100 µM GA3 phenotypically increasing seedling growth in both the Cheongcheong and salt-sensitive IR28 cultivars (**Figure 2C**). Here, we found that 100 µM GA3 significantly increased levels of all four bioactive GAs (GA1, GA3, GA4, and GA7) in the Cheongcheong, and IR28 cultivars compared to levels in both the control and NaCl-treated seedlings (**Figures 3A, B** **)**. When treated with 120 mM NaCl + 50 µM GA3, the Nagdong cultivar contained high levels of GA1, GA3, GA4, and GA7. The salt-tolerant Pokkali cultivar contained high levels of GA1, GA3 and GA7 when treated with 120 mM NaCl + 50 µM GA3 than treated with 120 mM NaCl only (**Figures 3C, D** **)**. Conversely, there was no significant difference between the levels of bioactive GAs observed in the control and 50 µM GA3-treated seedlings in the Nagdong and Pokkali cultivars (**Figures 3C, D** **)**.

### Effect of salinity on the expression of α-amylase and GA biosynthesis-related genes

**Figure 4:**
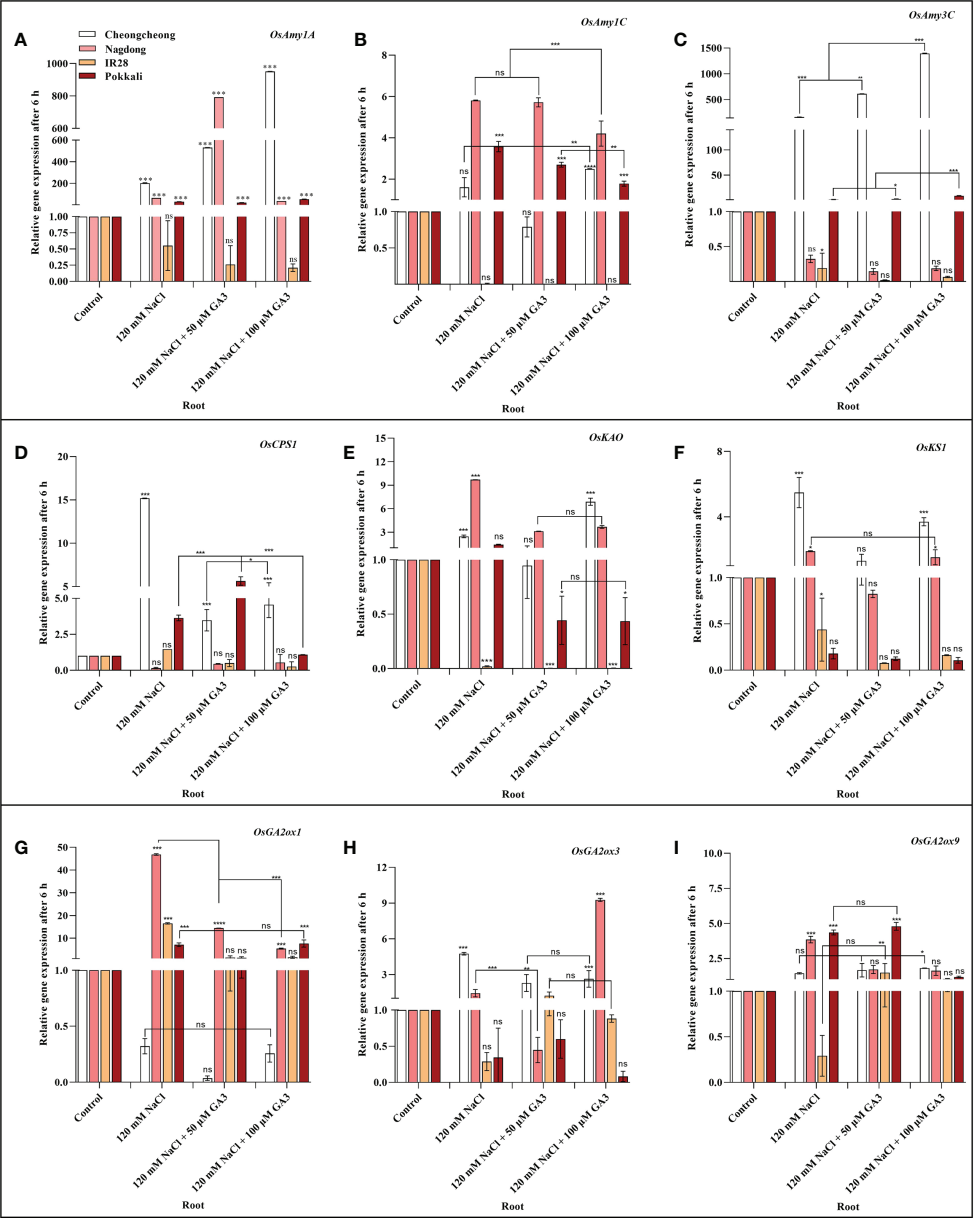
Effects of salt stress and exogenous GA3 application on α-amylase and GA metabolic gene expression in the roots of rice (*Oryza sativa* L.) seedlings **(A–I)**. After 14-days incubation in distilled water, rice seedlings were treated with distilled water (control), 120 mM NaCl, or 120 mM NaCl + 50 or 100 µM GA3. After 6 h incubation, gene expression levels in the roots of the different rice cultivars were analysed. Data are presented as the mean ± SE, with gene expression levels given relative to the expression levels of α-amylase genes in the control group. Asterisks indicate a significant difference compared to control, analysed by Tukey’s multiple comparisons test. *P < 0.05, **P < 0.01, ***P < 0.00; ns, not significant. GA, gibberellin; GA3, gibberellic acid.

**Figure 5:**
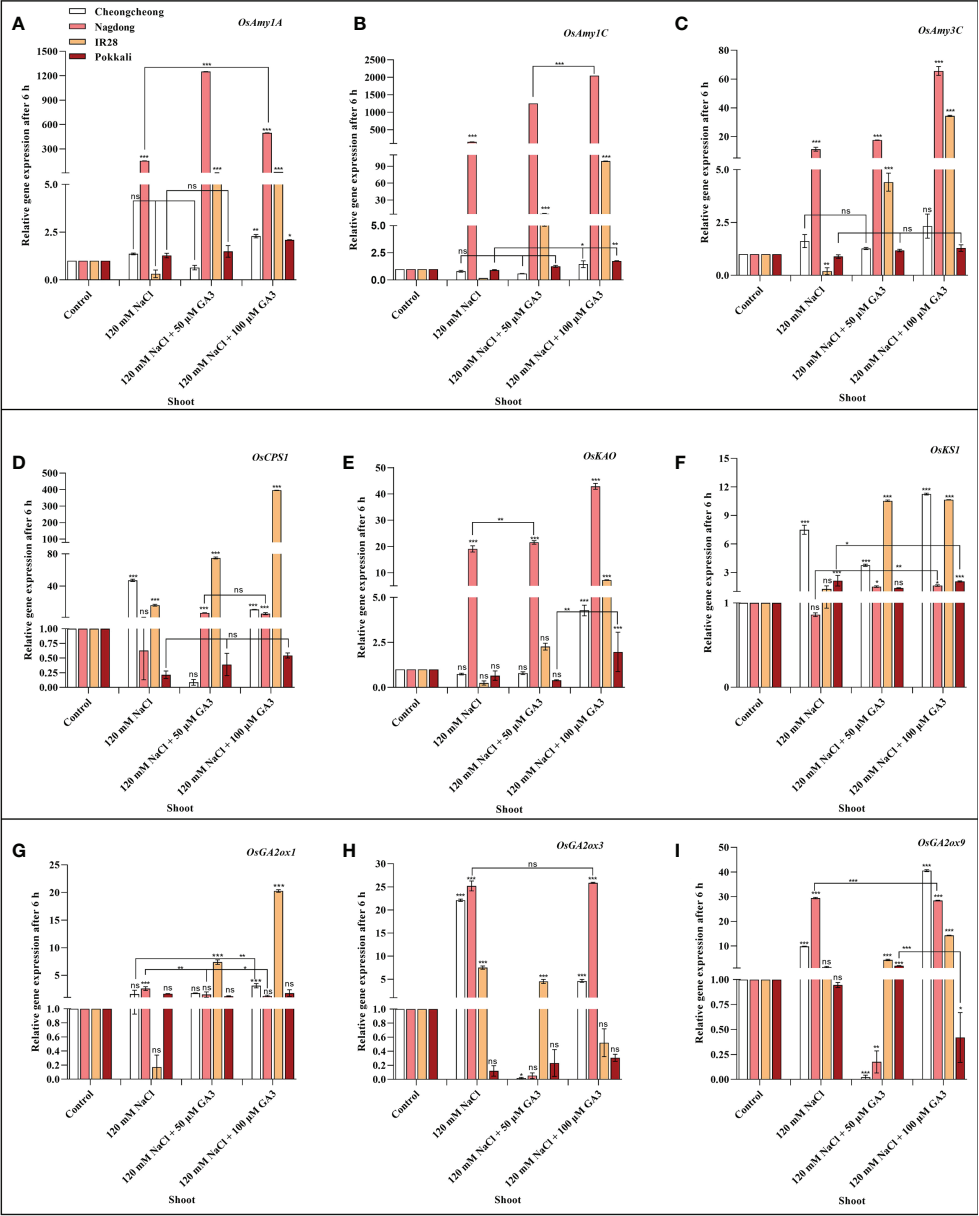
Effects of salt stress and exogenous GA_3_ application on α-amylase and GA metabolic gene expression in the shoots of rice (*Oryza sativa* L.) seedlings **(A–I)**. After 14-days incubation in distilled water, rice seedlings were treated with distilled water (control), 120 mM NaCl, or 120 mM NaCl + 50 or 100 µM GA3. After 6 h incubation, gene expression levels in the shoots of different rice cultivars were analysed. Data are presented as the mean ± SE, with gene expression levels given relative to the expression levels of α-amylase genes in the control group. Asterisks indicate a significant difference compared to control, analysed by Tukey’s multiple comparisons test. *P < 0.05, **P < 0.01, ***P < 0.00; ns, not significant. GA, gibberellin; GA3, gibberellic acid.

We next aimed to demonstrate how NaCl stress decreases bioactive GA content in rice cultivars. We thus analysed the induction of α-amylase and GA biosynthesis-related genes in rice seedlings under salt stress and treated with exogenous GA (**Figures 4A–I**, **Figures 5A** **–I**). After 6 h stress, we observed differential gene expression in all treatment conditions across the different rice cultivars compared with the control condition. The α-amylase-related genes, such as *OsAmy1A*, *OsAmy1C*, and *OsAmy3C*, help in starch degradation during seed germination. To investigate how salinity may increase or decrease the expression of α-amylase-related genes that are closely related to α-amylase-related production, the effects of NaCl and NaCl + 50 or 100 µM GA3 treatment on α-amylase gene expression were analysed. The results showed that after 6 h NaCl treatment, the expression levels of all α-amylase genes were significantly decreased in the roots and shoots of the salt-sensitive IR28 cultivar, whereas in the shoots of the Cheongcheong and Pokkali cultivars, no significant difference were observed compared to the control, Under NaCl stress, the α-amylase gene was significantly upregulated in the roots and shoots of the Nagdong cultivar compared to that in other cultivars. The exogenous application of 50 and 100 µM GA3 rescued the NaCl stress-induced decreases in α-amylase gene expression (**Figures 4**, **5**). Under salinity stress, 100 µM GA3 treatment significantly upregulated α-amylase-related genes in the roots and shoots of rice cultivars (**Figures 4A** **–C**, **5A** **–C**).

Bioactive GAs is regulated by biosynthetic and inactivation mechanisms. We, therefore, sought to understand how NaCl treatment reduced bioactive GA content by examining the effect of NaCl treatment and exogenous GA application on the expression of genes related to the biosynthesis and inactivation of GA; this analysis was performed in different rice varieties during the seedling stage. The expression profiles of GA biosynthesis genes demonstrated that NaCl treatment resulted in upregulated expression of *OsCPS1*, and *OsKS1* in the roots and shoots of the Cheongcheong cultivar compared to that of the other cultivars (**Figures 4D, F**, **5D, F** **)**. NaCl stress significantly downregulated *OsCPS1*, *OsKAO*, and *OsKS1* in the root of IR28. Conversely, *OsCPS1*, was upregulated in the shoots of IR28 and roots of the Pokkali cultivar (**Figures 4D**, **5D**). Salt stress, after 6 h incubation, upregulated *OsKAO* in the Nagdong roots and shoots compared to other cultivars. NaCl treatment differentially regulated the expression levels of *OsGA2oxi*, *OsGA2ox3*, and *OsGA2ox9* in the roots and shoots of different rice cultivars; NaCl stress upregulated *OsGA2ox1* in the roots, whereas *OsGA2ox3* and *OsGA2ox9* were upregulated in the shoots, of different rice cultivars (**Figures 4I, J**, **5I, J**). The results further showed that under 120 mM NaCl stress, 50 and 100 µM GA3 significantly upregulated *OsCPS1, OsKAO*, and *OsKS1* in the shoots of the Cheongcheong, IR28, and Nagdong cultivars (**Figures 5D** **–F**). Under NaCl stress, 50 and 100 µM GA3 significantly upregulated *OsGA2ox1* and *OsGA2ox9* in the shoots of the Cheongcheong and salt-sensitive IR28 cultivars (**Figures 5G, I**).

### Effect of salt stress and exogenous GA3 application on antioxidant activity

**Figure 6:**
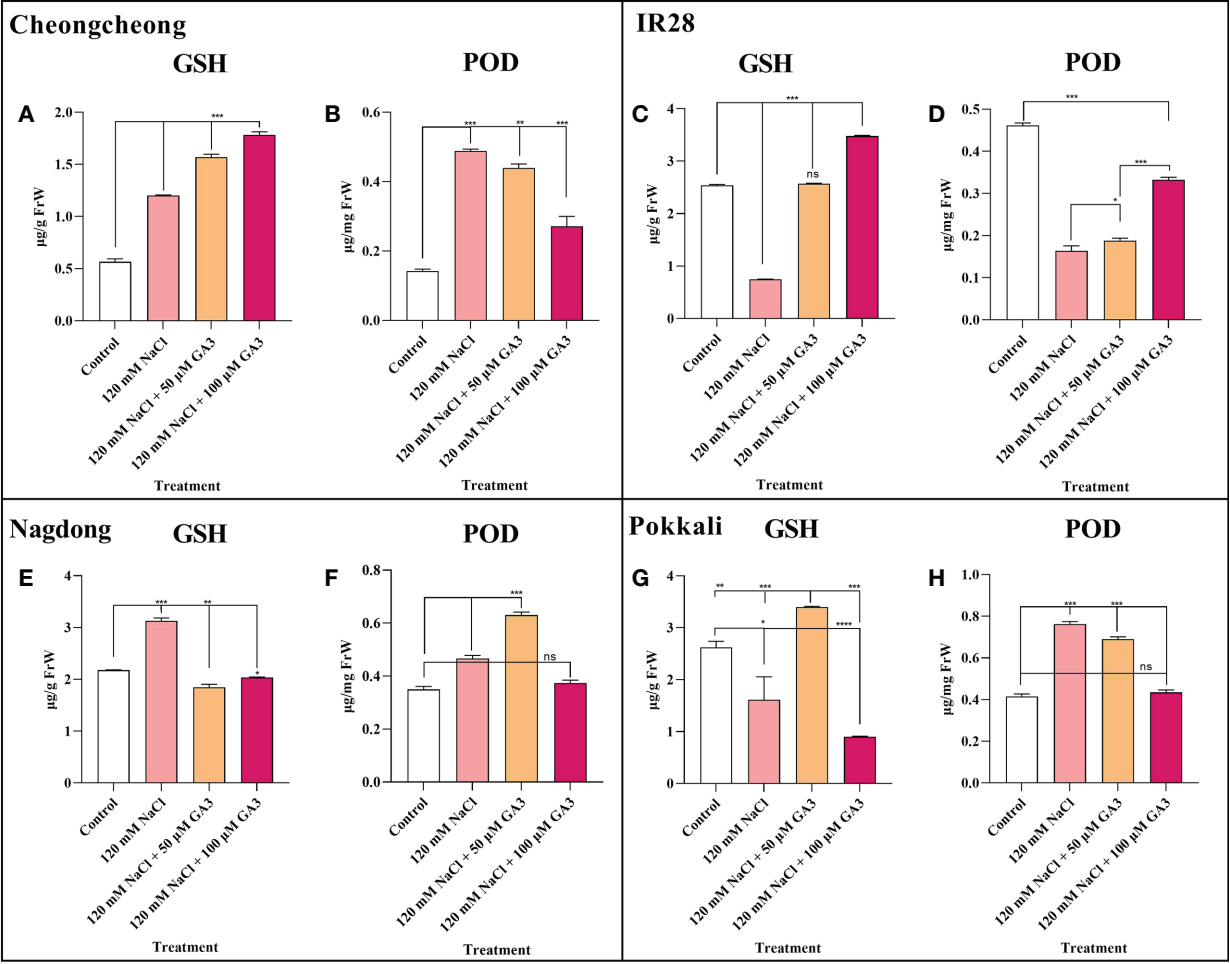
Effects of salt stress and exogenous GA3 application on antioxidant activity in rice (*Oryza sativa* L.) seedlings **(A–H)**. Antioxidant activity in 14-day-old rice seedlings treated with distilled water (control), 120 mM NaCl, or 120 mM NaCl + 50 or 100 µM GA3. Group differences were analysed by one-way ANOVA. Asterisks indicate a significant difference compared to control, analysed by Tukey’s multiple comparison test. *P < 0.05, **P < 0.01, ***P < 0.00; ns, not significant. FrW, frozen weight; GA3, gibberellic acid; GSH, glutathione; POD, peroxidase.

The activity of two vital antioxidants, GSH and POD, was analysed in different rice cultivar seedlings either under control conditions or treated with 120 mM NaCl ± exogenous GA3. We observed that compared to controls, 120 mM NaCl stress significantly increased GSH activity in the Cheongcheong and Nagdong cultivars (**Figures 6A, E** **)**. However, 120 mM NaCl stress significantly decreased GSH content in the salt-sensitive IR28, and the salt-tolerant Pokkali cultivars (**Figures 6C, G** **)**. In contrast, compared to the control group, other rice cultivars except the salt-sensitive IR28 cultivar showed significantly increased POD activity in response to NaCl stress (**Figures 6B, F, H** **)**. Exogenous GA3, at both 50 and 100 µM, could attenuate the effects of NaCl stress, significantly increasing GSH content in the rice seedlings of all cultivars, except the Nagdong cultivar (**Figures 6A, C, G** **)**. Similarly, 50 and 100 µM exogenous GA3 increased POD activity in Nagdong and IR28 seedlings compared to the activity in NaCl-only treated seedlings. However, in the Cheongcheong and Pokkali cultivars, there was no difference observed in POD activity with the additional application of exogenous GA3, compared to the 120 mM NaCl-only condition (**Figures 6B, H** **)**.

## Discussion

GAs play an energetic role in the enhancement of seed germination. It has been explored that GA biosynthesis is activated during seed germination (Ayele et al., 2012). During germination, GA content increases *via* the upregulation of GA biosynthesis and downregulation of GA inactivation (Yamauchi et al., 2004; Rieu et al., 2008). Inhibitors of GA biosynthesis stop seed germination, and exogenous GA reclaim these inhibitory effects (Gallardo et al., 2002). In addition, the *Arabidopsis ga1-3 mutan*t, and the *gib-1 mutant* of tomato are reported to be difficult to germinate without exogenous GA (Lee et al., 2002). Furthermore, it has been reported that an interaction effect of GA3 and salt, whereby high salt stress decreased germination percentage and seedling growth in sorghum by lowering GA3 levels (Ali et al., 2021). Our results indicated that NaCl stress significantly inhibited rice seed germination and their growth rate in different rice cultivars. However, these deleterious effects could be significantly recovered by 50 and 100 µM exogenous GA3 treatment (**Figures 2A** **–C**) These results infer that 120 mM NaCl stress may inhibit seed germination and seedling growth by lowering the GA content in rice cultivars (**Figures 3A** **–D**). According to Kobayashi et al., GA1 and GA4 are the principal bioactive forms of GA in rice (Kobayashi et al., 1988). In the current study, 120 mM NaCl stress significantly decreased the levels of bioactive GA3, GA4, and GA7 in both Cheongcheong and Nagdong cultivars compared to controls, whereas salt stress downregulated levels of bioactive GA1, GA3, and GA7 in the salt-sensitive IR28 and salt-tolerant Pokkali cultivars (**Figures 3A** **–D**). These results showed that salinity inhibits rice germination by decreasing bioactive GA content. A previous study reported that salinity induced bioactive GA deficiency but the specific forms affected could not be distinguished at that time (Kim et al., 2006). In the current study, salt stress decreased the bioactive GA content, which inhibited seed germination in rice cultivars. We believe that bioactive GA3, GA4, and GA7 may be implicated in the decline of rice seed germination and seedling growth in the various rice genotypes (**Figures 3A** **–D**). In the current study, the deficiencies in bioactive GAs were significantly reversed by exogenous GA3 (**Figures 3A** **–D**).

A previous study described that in rice, α-amylase genes such as *OsAmy1A*, *OsAmy1C*, *OsAmy3C*, and *OsAmy3E* were favourably upregulated by GA in wild-type seeds, apart from the GA receptor mutant *gid1* (a null mutant for GA INENSITIVE DWARF1). In seeds from the GA signalling repressor mutant *slr1*(a null mutant for Della), α-amylase gene expression levels were similar to those of GA-treated wild-type seeds, regardless of GA treatment (Yano et al., 2015). This study corroborates the finding that GA controls α-amylase activity through transcriptional regulation in rice. Our results indicated that NaCl treatment significantly downregulated *OsAmy1C* and *OsAmy3C* in the shoots of IR28; under this salt stress, other cultivars also expressed these genes differentially in the root and shoot regions. Furthermore, NaCl treatment decreased the bioactive GA content in different rice cultivars compared to the controls (**Figures 3A** **–D**). In the current study, it was difficult to correlate or specify the decreased bioactive GAs with α-amylase gene expression; however, this up- and downregulation may be due to the different varietal responses to salinity. In contrast, bioactive GA content and α-amylase genes were significantly upregulated by 50 and 100 µM GA3 treatment compared to that by both controls and seedlings treated with 120 mM NaCl (**Figures 3A–D**, **4A–C**, **5A** **–C**). A previous study reported that the initiation of GA biosynthesis in the scutellum is closely associated with the induction of α-amylase genes in the scutellar epithelium (Appleford and Lenton, 1997). In the recent study, we also found a high expression of α-amylase genes in different salt-stressed rice cultivars treated with exogenous GA3. Therefore, exogenous GA3 initiates GA biosynthesis in the roots of rice scutellum, which may induce α-amylase genes in the different rice cultivars (**Figures 4A** **–C**, **5A** **–C**).

Bioactive GA content is collectively controlled by biosynthetic and inactivation processes. Some of genes in rice such as, *CPS*, *KAO*, *KO*, *KS*, *GA2ox*, and *GA3ox* catalyse GA biosynthesis, and GA2ox can inactivate bioactive GAs (Dill and Sun, 2001; Olszewski et al., 2002; Hedden and Thomas, 2012). To investigate the mechanism by which NaCl stress decreases bioactive GA content, the effects of 120 mM NaCl and exogenous GA3 treatment on the expression of genes linked to the biosynthesis and inactivation of bioactive GAs were examined. Our results identified that 14-day-old rice seedlings show differential expression of GA biosynthetic genes in rice cultivars after 6 h salt stress. The most substantial upregulation of GA biosynthetic genes induced by NaCl treatment was in the roots and shoots of Cheongcheong and Nagdong cultivars (**Figures 4D, I**, **5D, I** **)**. Similarly, these genes were significantly upregulated by treatment with NaCl and exogenous GA3 in the shoots of the salt-sensitive IR28 cultivar (**Figures 5D, F, G, I** **)**. Previous studies have reported that *GA2ox* genes, upregulated by NaCl treatment, are responsible for regulating rice seed germination (Lin et al., 2008; Liu et al., 2018). Our study suggests that the bioactive GA content was declined by NaCl treatment (**Figures 3A** **–D**), indicating that 120 mM NaCl triggers bioactive GA deficiency by boosting bioactive GA inactivation (**Figure 1**), alternatively by inhibiting bioactive GA biosynthesis. High induction of GA biosynthetic genes may be an effect of the negative feedback regulation occurring as a consequence of NaCl-induced bioactive GA deficiency. This study is similar to those in *Arabidopsis thaliana*, a dicotyledonous typical plant that also counter to high salt stress with a decrease in endogenous GA content and upregulation of *GA2ox* gene expression (Magome et al., 2008). In the current study, we found that under 120 mM NaCl stress, *OsGA2ox3* was significantly upregulated in entire rice cultivars, except the salt-tolerant Pokkali cultivar (**Figure 5H**); this may be the reason why salinity decreased bioactive GA content. A previous study reported that through soybean seed germination, salt stress declined bioactive GA content by undesirably regulating GA biosynthesis (Shu et al., 2017). Here, we conclude that under salt stress, exogenous GA3 plays an important role in reducing upregulated GA biosynthetic genes. Similarly, the downregulated GA biosynthetic genes can be upregulated by exogenous GA3 (**Figures 4D–I**, **5D** **–I**).

Previous investigations have shown that NaCl treatment causes cellular injury due to oxidative stress (Liang et al., 2003; Zhu, 2016). The cellular oxidative pressure is further correlated with seed germination activity and seedling growth (Adamus-Białek et al., 2017).

A recent study suggests that a balanced redox status in rice would be the consequence of a synergetic activity of both enzymatic and non-enzymatic antioxidant mechanisms, resulting in tolerance to Pb-induced oxidative damage (Khan et al., 2021). Under oxidative stress, excessive ROS accumulation damages plant tissues (Jaskulak et al., 2018). A previous study reported that GA3 application under salt stress improved the antioxidant activity in maize seedlings (Shahzad et al., 2021). Our study also indicated that exogenous GA significantly increased the antioxidant activity in different rice cultivars (**Figures 6A, C, D, F, G** **)**. However, we suggest that exogenous GA3 does not significantly upregulate all antioxidant activity. In the present study, we noticed that under, salt stress with exogenous GA3 application, different rice genotypes differentially regulated antioxidant activity (**Figure 6**). A previous study suggested that salt stress decreased POD and SOD activity in the salt-sensitive Zhegeng 99 rice cultivar (**Figure 6**). In the current study, we found that salt stress significantly decreased GSH and POD activity in the salt-sensitive IR28 cultivar, which could be significantly rescued by exogenous GA3 (**Figures 6C, D** **)**.

## Conclusions

Global agriculture is at greater risk from crop loss due to soil salinization, particularly for rice. The current Petri dish-based investigation showed the impact of 120 mM NaCl treatment along with exogenous GA3 in various rice cultivars. We found that rice cultivars germination rates, seedling, and GA content were all considerably reduced by 120 mM NaCl stress. The expression of genes linked to GA production and inactivation, antioxidants, and α-amylase was also observed to change amongst rice genotypes during salt stress. In the present study, we found that 50 and 100 µM GA3 significantly alleviated the effects of 120 mM NaCl stress among rice genotypes. According to findings from the current study, salt-tolerant Pokkali and Nagdong cultivars should be treated with 50 µM GA3 and salt-sensitive IR28 and Cheongcheong cultivars should be treated with 100 μM GA3. The current study provided evidence that the exogenous GA3 application can significantly rescue the salinity effect, and the rice can be grown environmentally friendly near coastal areas.

## Data availability statement

The raw data supporting the conclusions of this article will be made available by the authors, without undue reservation.

## Author contributions

MF planned, designed, and performed the study, analysed the data, and wrote the findings. MK helped with GAs analysis, and D-DZ helped with data collection. SA helped with the antioxidant analysis. E-GK, Y-HJ, J-RP contributed to the experimental resources, I-JL and K-MK drafted and edited the manuscript. All authors contributed to the article and approved the submitted version.

## Funding

This work was supported by a National Research Foundation of Korea grant funded by the Korean Government (NRF-2021M3E5E6022715).

## Conflict of interest

The authors declare that the research was conducted in the absence of any commercial or financial relationships that could be construed as a potential conflict of interest.

## Publisher’s note

All claims expressed in this article are solely those of the authors and do not necessarily represent those of their affiliated organizations, or those of the publisher, the editors and the reviewers. Any product that may be evaluated in this article, or claim that may be made by its manufacturer, is not guaranteed or endorsed by the publisher.
